# Climate variability amplifies the need for vector-borne disease outbreak preparedness

**DOI:** 10.1073/pnas.2507311122

**Published:** 2025-08-18

**Authors:** William S. Hart, James W. Hurrell, Alexander R. Kaye, Meera Chand, Matt J. Keeling, Robin N. Thompson

**Affiliations:** ^a^Wolfson Centre for Mathematical Biology, Mathematical Institute, Mathematical, Physical and Life Sciences Division, University of Oxford, Oxford OX2 6GG, United Kingdom; ^b^Department of Atmospheric Science, Colorado State University, Fort Collins, CO 80521; ^c^Zeeman Institute for Systems Biology and Infectious Disease Epidemiology Research, Faculty of Science, Engineering and Medicine, University of Warwick, Coventry CV4 7AL, United Kingdom; ^d^Mathematics Institute, Faculty of Science, Engineering and Medicine, University of Warwick, Coventry CV4 7AL, United Kingdom; ^e^UK Health Security Agency, Canary Wharf, London E14 4PU, United Kingdom; ^f^Department of Life Sciences, Faculty of Science, Engineering and Medicine, University of Warwick, Coventry CV4 7AL, United Kingdom

**Keywords:** climate variability, climate-sensitive infectious disease, vector-borne disease, public health policy

## Abstract

In locations that do not currently experience vector-borne disease (VBD) outbreaks but may be at risk under climate change, modeling future climate suitability for transmission is important for outbreak preparedness. Uncertainty in the future climate arises from three sources—differences in emissions scenarios, structural uncertainty across climate models, and internal climate variability (ICV)—but ICV is rarely considered in climate-VBD studies. Here, we demonstrate that ICV is a key source of uncertainty in climate suitability for VBD transmission, even decades into the future. Because of ICV, suitable climate conditions for transmission may arise in many locations sooner than expected under climate change alone.

Climate change is altering the dynamics of vector-borne diseases (VBDs) such as dengue, which is transmitted by *Aedes* mosquitoes. *Aedes* populations are expanding in North America and Europe, with local dengue transmission reported near both Los Angeles and Paris for the first time during 2023. This raises concerns for locations including the United Kingdom, where *Aedes albopictus* has been detected but is not established (1). To quantify future outbreak risks, climate projections have been coupled with vector and pathogen dynamics models (2–4).

Climate projections derived from general circulation models (GCMs) are subject to three uncertainty sources. Internal climate variability (ICV) refers to natural fluctuations that occur irrespective of external factors like greenhouse gas emissions (5, 6), generating aleatory uncertainty because of the chaotic nature of the climate (Fig. 1*A*). Large (single-model) ensembles of simulations, using slightly different initial conditions in each simulation, are important for quantifying ICV (6). Model uncertainty (which is epistemic) arises from differences in formulation between GCMs (5) and is often quantified using multimodel ensembles (Fig. 1*B*), e.g., the Coupled Model Intercomparison Project (CMIP) (7). Finally, scenario uncertainty (also epistemic) relates to possible future pathways of external climate forcing, including greenhouse gas emissions (5), and is analyzed by running GCMs under different scenarios (Fig. 1*C*), e.g., the Shared Socioeconomic Pathways (SSPs) in CMIP6 (8).

**Fig. 1.**
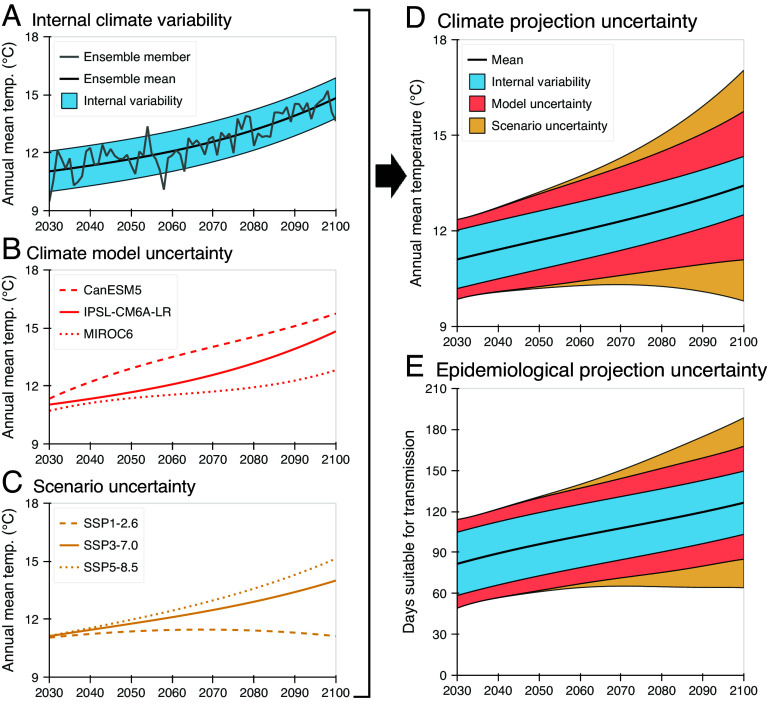
Sources of uncertainty in climate projections, and corresponding uncertainty in climate-VBD projections. (*A*) Internal climate variability (ICV). Annual mean temperature projections for London from one simulation of the IPSL-CM6A-LR GCM under SSP3-7.0 (gray curve), theoretical single-model ensemble mean (black curve), and 90% prediction interval (PI) characterizing uncertainty due to ICV (blue shaded region). (*B*) Model uncertainty. Theoretical single-model ensemble mean projections for three GCMs under SSP3-7.0. (*C*) Scenario uncertainty. Theoretical multimodel ensemble mean projections from ten GCMs for SSP1-2.6 [“sustainability” (8)], SSP3-7.0 [“regional rivalry” (8)] and SSP5-8.5 [“fossil-fueled development” (8)]. (*D*) Mean projections over ten GCMs and three scenarios, and 90% PI, decomposed into the contribution from ICV (averaged over models/scenarios; blue), and additional contributions from model uncertainty (averaged over scenarios; red) and scenario uncertainty (orange). (*E*) Equivalent uncertainty decomposition for projections of the annual number of days for which temperatures permit dengue transmission by *Aedes albopictus*.

While model and scenario uncertainty are often considered in climate-VBD studies, ICV is usually neglected (*SI Appendix*, Literature search). Here, we apply methods for partitioning climate uncertainty in the context of future VBD risks, showing that ICV can lead to suitable climate conditions for outbreaks sooner than expected under climate change alone.

## Results

Considering daily temperature projections for London in 2030–2080 from ten CMIP6 GCMs and three SSPs (9), we partitioned uncertainty in annual mean temperatures into contributions from ICV, model uncertainty, and scenario uncertainty (Fig. 1*D*). Since a single realization was available for each model and scenario pair, similarly to (5), we fitted (here, cubic) polynomials to estimate single-model and scenario ensemble means (i.e., forced responses to climate change) and the extent of ICV (assumed time-independent). We focused on the average uncertainty due to ICV over models and scenarios; however, estimates of the interannual variance [in ${{(}^{\circ }C)}^{2}$] due to ICV vary from 0.25 to 0.58 between models (mean 0.35) under SSP1-2.6, 0.22 to 0.41 (0.30) under SSP3-7.0, and 0.21 to 0.37 (0.28) under SSP5-8.5.

Then, using a model of temperature suitability for *Ae. albopictus*-borne dengue transmission (10), we projected the annual number of days suitable for transmission (Fig. 1*E*) and again partitioned uncertainty arising from the three climate uncertainty sources. Notably, ICV is responsible for a substantial proportion of uncertainty in the number of suitable days, even decades into the future; e.g., in 2080, 38% of the prediction interval (PI) for the annual mean temperature is attributable to ICV, compared to 48% for days suitable for transmission, consistent with previous findings regarding uncertainty in nonlinear climate-dependent impacts (11).

**Fig. 2.**
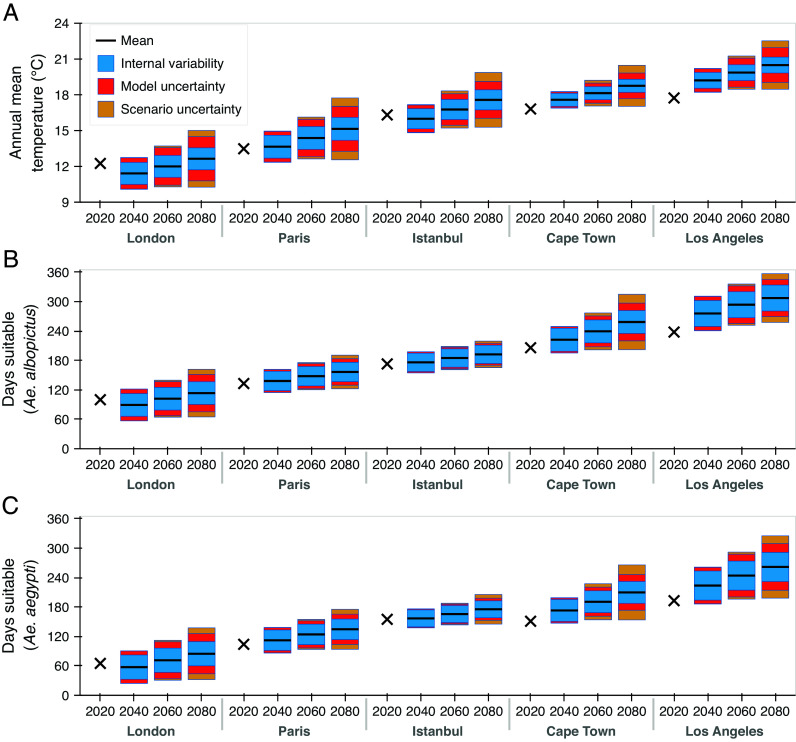
Uncertainty in different locations and for transmission by different vector species. (*A*) Annual mean recorded temperature in 2020 (black crosses) (12); and projections for 2040, 2060, and 2080, averaged over ten GCMs and three SSPs (black horizontal lines), alongside 90% PIs (bars) decomposed into contributions from ICV (averaged over models/scenarios; blue), and from additional model uncertainty (averaged over scenarios; red) and scenario uncertainty (orange). Results are shown for London, Paris, Istanbul, Cape Town, and Los Angeles. (*B*) Corresponding results for the annual number of days for which temperatures permit *Ae. albopictus*-borne dengue transmission, using the suitability model from (10). (*C*) Equivalent results to (*B*), except considering *Ae. aegypti*-borne dengue transmission.

We conducted equivalent analyses using data from five cities that do not currently experience sustained dengue transmission but may be at future risk (Fig. 2), considering temperature-suitability models for *Ae. albopictus*-borne (Fig. 2*B*) or *Ae. aegypti*-borne (Fig. 2*C*) dengue transmission (10). Additionally, we evaluated transmission suitability using recorded 2020 weather data (12) (black crosses in Fig. 2). Precise uncertainty contributions differ between cities, as might be expected given their different climate conditions. Nonetheless, for each location, ICV contributes a substantial proportion of uncertainty in transmission-suitability projections. Moreover, year-to-year temperature fluctuations associated with ICV accelerate elevated suitability; e.g., if only model and scenario uncertainty are considered (i.e., theoretical single-model ensemble means are used for each model and scenario pair), the annual risk of ≥120 d of suitability for dengue transmission by *Ae. albopictus* in London first exceeds 5% in 2047, compared to 2038 when ICV is also considered (Fig. 1*E*).

## Discussion

Despite usually being neglected in climate-VBD studies, ICV contributes substantial uncertainty to VBD risk projections and could generate years with high climate suitability for transmission. Concerns have been raised that policy responses to VBD risks have been inadequate [e.g., the third UK Climate Change Risk Independent Assessment concluded that “more action (is) needed” to mitigate VBD risks (13)]. Our findings amplify the need for outbreak preparedness. In locations without established vector populations, vector surveillance is crucial, particularly at points of entry. Following vector detection, controls to prevent or delay establishment, such as breeding site removal, are essential. When vectors establish, additional requirements include clinical testing for VBDs, public information campaigns, and outbreak response plans.

Quantitative VBD risk estimates are important for targeting vector and clinical surveillance, requiring collaboration between climate scientists, ecologists, epidemiologists, and policy specialists. Here, we used simple temperature-suitability models to demonstrate that it is crucial to consider ICV in climate-VBD projections. However, policy-informing models should consider additional climate variables (e.g., precipitation and humidity) and nonclimate factors (e.g., human population densities and intervention impacts), and uncertainty about how all climate and other factors affect VBD dynamics. The development of accessible software tools to guide policy planning is also key (14).

Adapting methodology from a seminal climate science article (5), our analyses involved assumptions. We fitted cubic polynomials to estimate (single-model) ensemble mean values from single climate realizations for each model and scenario pair. We verified the robustness of our qualitative results to this choice of fitted curves, obtaining similar results using cubic splines (*SI Appendix*, *Partitioning Climate Projection Uncertainty*). We weighted the climate model and scenario pairs uniformly when partitioning uncertainty and assumed that the extent of ICV is time-invariant. These assumptions could be relaxed by employing more complex methods. Use of large ensemble climate projections could facilitate estimation of temporal variations in ICV (6), although assessing how anthropogenic climate change will affect future ICV remains a key challenge in climate science. However, this was not necessary to communicate our main message: ICV should be considered alongside model and scenario uncertainty when generating climate-sensitive VBD projections.

In summary, ICV may lead to suitable climate conditions for VBD transmission in many locations sooner than expected under climate change alone. As the World Health Organization Working Group on Health in Climate Change advises, proactive measures are needed to mitigate the health impacts of climate change and to have “zero regrets” (15).

## Materials and Methods

Daily temperature projections (2030–2100) were obtained from the Inter-Sectoral Impact Model Intercomparison Project phase 3b (9). Recorded weather station temperature data for 2020 were retrieved using the Meteostat Python library (12). Temperature data were combined with temperature-suitability models for dengue transmission by *Ae. albopictus* and *Ae. aegypti* (10) to project the annual number of days suitable for transmission. Uncertainty in projections was partitioned adapting the approach of (5). For details, see the SI Text.

## Supplementary Material

Appendix 01 (PDF)

## Data Availability

Data and code are available at https://github.com/idm-oxford/climate-vbd-uncertainty-paper (archived at https://doi.org/10.5281/zenodo.15318576). We provide an online software application to visualize uncertainty in climate-VBD projections in different locations at https://idm-oxford.github.io/climate-vbd-uncertainty-paper/. Previously published data were used for this work (9).
