# Supplementary material for: Climate variability amplifies the need for vector-borne disease outbreak preparedness
Source: Proc Natl Acad Sci U S A. 2025 Aug 18;122(34):e2507311122. doi: 10.1073/pnas.2507311122 (PMC12403081; doi:10.1073/pnas.2507311122)
Supplement: Supplementary file 1 — Appendix 01 (PDF) [file pnas.2507311122.sapp.pdf]

PNAS

Supporting Information for

Climate variability amplifies the need for vector-borne disease outbreak preparedness

W.S. Hart, J.W. Hurrell, A.R. Kaye, M. Chand, M.J. Keeling, R.N. Thompson

Corresponding author: William S. Hart
Email: william.hart@maths.ox.ac.uk

This PDF file includes:

Supporting text
SI references

Supporting Information Text

Literature search

We searched PubMed for articles published in English from database inception to 7 May 2025, with the following search terms: ("climate change") AND (vector) AND (model) AND ((uncertainty) OR ("climate variability") OR ("climate change scenarios") OR (SSPs)). This returned 239 articles, of which 110 reported projections relating to the impact of climate change on VBDs or their associated vectors. Of these 110 papers, 91 studies presented projections under multiple climate change scenarios (i.e., scenario uncertainty), 30 studies presented projections from multiple climate models and/or explicit measures of spread or uncertainty between models (i.e., model uncertainty) and only three studies (1–3) considered uncertainty due to ICV (defined as analysing uncertainty in climate outcomes at one or more specific time points for an individual model/scenario pair). None of the studies analysed the combined impact of all three uncertainty sources.

A spreadsheet with the results of the literature search is available at https://github.com/idm-oxford/climate-vbd-uncertainty-paper.

Climate projection data

Near-surface daily mean temperature projections (bias-corrected and statistically downscaled to a 0.5°x0.5° global grid) were obtained from the Inter-Sectoral Impact Model Intercomparison Project (ISIMIP) phase 3b (4). We used temperature projection data for 2030-2080 for grid squares corresponding to London (UK), Paris (France), Istanbul (Turkey), Cape Town (South Africa) and Los Angeles (USA) in our analyses. Data were available for ten CMIP6 GCMs (CanESM5, CNRM-CM6-1, CNRM-ESM2-1, EC-Earth3, GFDL-ESM4, IPSL-CM6A-LR, MIROC6, MPI-ESM1-2-HR, MRI-ESM2-0, UKESM1-0-LL) for each of three scenarios (SSP1-2.6, SSP3-7.0 and SSP5-8.5). A single simulation was available for each model/scenario pair.

Recorded weather data

Daily mean temperature data from 2020 were obtained from weather stations in the vicinity of each city (London, Paris, Istanbul, Cape Town and Los Angeles) using the Meteostat Python library (5).

Partitioning climate projection uncertainty

Since only a single model simulation was available for each GCM/SSP combination, we used a similar approach to Hawkins and Sutton (6) to: i) estimate the extent of year-to-year temperature variations due to ICV in each simulation, and ii) partition uncertainty into estimated contributions from ICV, climate model uncertainty and scenario uncertainty.

In detail, in our main analyses we fitted cubic polynomials to the annual mean temperature time series (calculated from the daily temperature data) for each simulation using ordinary least squares estimation. The annual mean temperature, $T_{s,m,t}$, for scenario s and model m in year t could then be written as

$$T_{s,m,t} = p_{s,m,t} + \varepsilon_{s,m,t},$$

where $p_{s,m,t}$ is the value of the model/scenario-specific fitted polynomial in year t and $\varepsilon_{s,m,t}$ is the residual term (assumed to be drawn from a normal distribution with mean 0 and variance ${\sigma_{s,m}}^2$). The smooth fitted polynomial was assumed to represent the forced response to climate change under that model/scenario pair (i.e., the ensemble mean that would be obtained from a large ensemble of simulations of the same climate model with identical external forcing (e.g., from greenhouse gases), but slightly different initial conditions), and the residual terms to characterise year-to-year variations around this forced response due to ICV. We note that this formulation involves an assumption that the extent of ICV remains constant over time. We also conducted alternative analyses in which we fitted cubic splines instead of polynomials to each time series (using the SciPy “make_smoothing_spline” function), finding that the exact choice of fitted curve has only a small effect on the estimated relative contributions of different climate uncertainty sources (for example, using splines under the default settings from the “make_smoothing_spline” function, an estimated 37% of the width of the PI for the annual mean temperature in London in 2080 is attributable to ICV, compared to an estimate of 38% using cubic polynomials).

We then partitioned the total variance, V_t, of the temperature projections, $T_{s,m,t}$, at time t (where this variance is calculated over uncertainty in the climate model, m, and the scenario, s, as well as randomness in the residual terms, $\varepsilon_{s,m,t}$, associated with ICV) as follows. By the law of total variance,

$$\begin{aligned} V_t &= \mathrm{E}_{s,m}\left[\mathrm{Var}\left(T_{s,m,t} \mid s,m\right)\right] + \mathrm{Var}_{s,m}\left(\mathrm{E}\left[T_{s,m,t} \mid s,m\right]\right) \\ &= \mathrm{E}_{s,m}\left[{\sigma_{s,m}}^2\right] + \mathrm{Var}_{s,m}\left(p_{s,m,t}\right), \end{aligned} \tag{S1}$$

where for clarity we write $\mathrm{E}_{s,m}$ and $\mathrm{Var}_{s,m}$ to represent means and variances, respectively, taken over

scenarios, s, and models, m. Here, $V^{(internal)} = \mathrm{E}_{s,m}\left[\sigma_{s,m}{}^2\right]$ is the component of the variance associated with ICV (i.e., the variance due to ICV under individual model/scenario pairs, $\sigma_{s,m}{}^2$, averaged over models/scenarios), and $\mathrm{Var}_{s,m}(p_{s,m,t})$ gives the variance of the forced response, which is associated with a combination of climate model and scenario uncertainty. We note, again following Hawkins and Sutton (6), that (consistent with the assumptions made when fitting cubic polynomials to the data) we assumed the variance component due to ICV, $V^{(internal)}$, to be time independent. Since the residual variance terms, $\sigma_{s,m}{}^2$, characterising the extent of ICV under individual models/scenarios were unknown, we approximated these by temporal means of the squared residual terms,

$$\sigma_{s,m}{}^2 \approx \mathrm{E}_t\left[\varepsilon_{s,m,t}{}^2\right].$$

This choice of variance estimate reflects an assumption of known forced responses, $p_{s,m,t}$ (i.e., we did not use unbiased estimates of $\sigma_{s,m}{}^2$ that account for estimation of the forced responses). The rationale for this choice is that we used the estimates of $\sigma_{s,m}{}^2$ to determine prediction intervals relative to the model/scenario-averaged estimated forced response, as described below. We note that instead using unbiased estimates of $\sigma_{s,m}{}^2$ would have led to an increased estimate of the extent of ICV.

Applying the law of total variance to the second term of equation (S1) then gives

$$V_t = V^{(internal)} + \mathrm{E}_s\left[\mathrm{Var}_m(p_{s,m,t})\right] + \mathrm{Var}_s\left(\mathrm{E}_m\left[p_{s,m,t}\right]\right).$$

Here, $V_t{}^{(model)} = \mathrm{E}_s\left[\mathrm{Var}_m(p_{s,m,t})\right]$ is the component of the forced response variance due to climate model uncertainty (i.e. the variance in the forced response across models for each scenario, averaged over scenarios). Then, $V_t{}^{(scenario)} = \mathrm{Var}_s\left(\mathrm{E}_m\left[p_{s,m,t}\right]\right)$ gives the remaining variance, which is due to scenario uncertainty in the model-averaged forced response. We note that an asymmetry in the expressions for different variance components arises from the sequential treatment of uncertainty sources; while in principle the sources could be considered in a different order, we chose to use the same decomposition as Hawkins and Sutton (6).

Finally, we generated partitioned 90% PIs by first assuming annual mean temperature observations to be drawn from a normal distribution with mean equal to the model/scenario-averaged forced response, $\mathrm{E}_{s,m}(p_{s,m,t})$. Then, 90% PIs corresponding to normally distributed random variables with variances $V^{(internal)}$, $V^{(internal)} + V_t{}^{(model)}$ and V_t were computed to characterise the contribution of ICV, the combined contribution of ICV and model uncertainty (so the difference between this interval

and the first represents additional uncertainty due to model uncertainty), and the overall uncertainty incorporating all three uncertainty sources, respectively. We emphasise that these PIs are intended only to illustrate the relative amount of uncertainty contributed by each source, rather than to give specific ranges of values in which the sources of uncertainty individually make the temperature likely to lie – for example, the blue shaded region in Fig. 1D does not necessarily reflect the likely range of temperature values under any specific individual model/scenario combination.

Temperature suitability for dengue virus transmission by *Aedes* vectors

As an illustrative example, we assumed that temperatures in the range 16.2-31.6℃ are suitable for dengue virus transmission by *Ae. albopictus* and temperatures in the range 17.8-34.6℃ are suitable for dengue virus transmission by *Ae. aegypti*. These ranges were derived by Mordecai *et al.* (7) based on a mechanistic host-vector transmission model. In that model, temperature-dependent parameters correspond to traits including vector life cycle characteristics and the extrinsic incubation period of infected vectors. Mordecai *et al.* (7) used Bayesian methods to infer the temperature dependence of individual model parameters from laboratory data. The quoted temperature bounds then correspond to posterior means of the minimum and maximum temperatures at which any transmission is possible.

Combining the temperature projection data with these suitability ranges, we calculated (under the climate simulations for each GCM and SSP) the portion of each year with suitable daily mean temperatures for transmission. We characterised and partitioned uncertainty in the annual number of days suitable arising due to ICV, climate model uncertainty and scenario uncertainty, using the same method as described for the temperature data above.

SI references

1. K. Hayashi, M. Fujimoto, H. Nishiura, Quantifying the future risk of dengue under climate change in Japan. *Front. Public Health* **10** (2022).
2. A. R. Kaye, *et al.*, The impact of natural climate variability on the global distribution of Aedes aegypti: a mathematical modelling study. *Lancet Planet. Health* **8**, e1079–e1087 (2024).
3. A. J. Monaghan, *et al.*, The potential impacts of 21st century climatic and population changes on human exposure to the virus vector mosquito Aedes aegypti. *Clim. Change* **146**, 487–500 (2018).
4. S. Lange, M. Büchner, ISIMIP3b bias-adjusted atmospheric climate input data. https://doi.org/10.48364/ISIMIP.842396.1. Deposited 2021.
5. Meteostat. Available at: https://meteostat.net/en/ [Accessed 22 January 2025].

6. E. Hawkins, R. Sutton, The potential to narrow uncertainty in regional climate predictions. *Bull. Am. Meteorol. Soc.* **90**, 1095–1108 (2009).

7. E. A. Mordecai, *et al.*, Detecting the impact of temperature on transmission of Zika, dengue, and chikungunya using mechanistic models. *PLoS Negl. Trop. Dis.* **11**, e0005568 (2017).
